# Impact of urinary diversion on survival in locally advanced cervical carcinoma with obstructive uropathy in Tanzania

**DOI:** 10.3332/ecancer.2025.1940

**Published:** 2025-07-01

**Authors:** Gemini L Shayo, Latifa Rajab Abdallah, Emanuel L Lugina

**Affiliations:** 1 Muhimbili University of Health and Allied Sciences (MUHAS), Dar es Salaam, Tanzania; 2 Ocean Road Cancer Institute (ORCI), Dar es Salaam, Tanzania

**Keywords:** cervical cancer, percutaneous nephrostomy, obstructive uropathy, urinary diversion, overall survival, kidney function

## Abstract

**Background:**

Locally advanced cervical cancer (LACC) can result in obstructive uropathy (OU). Urinary diversion (UD) is the treatment of LACC patients with OU. This study assessed the benefits of UD before or during radiotherapy by examining its effect on improving kidney function and survival in patients with LACC.

**Materials and methods:**

The study retrospectively analysed the clinical data of 119 women with LACC treated from January 2020 to December 2021. The treatment intention (radical or palliative) was decided by a multidisciplinary team based on the disease stage, Karnofsky performance status and degree of renal derangement. Treatment and outcome details were retrieved from electronic records. This included obtaining serum creatinine levels before the UD, 7 days after, 21 days after and 1 month after the UD. Time to normalisation of serum creatinine, feasibility of delivering planned treatment and overall survival were determined. The impact of various prognostic factors on outcomes was determined using univariate or multivariate analysis. The significance level was set at 0.05.

**Results:**

The mean age was 51.1 ± 9.9 years. Approximately a third of patients underwent UD. Percutaneous nephrostomy was the most frequently performed type of UD (92%). About 85% of patients had hydronephrosis, and 56.3% had unilateral hydronephrosis. The mean baseline serum creatinine level was 662 µmol/L for the entire cohort. There was a 53% reduction of serum creatinine from the baseline to 30 days post-UD (*p* = 0.001). The median equivalent dose in 2-Gy (EQD2) for the whole cohort was 86 Gy. The median survival time for the entire cohort was 20 months. In the multivariate analysis, UD resulted in a 40% decreased mortality risk (aHR 0.6, *p*-value = 0.03). Patients who did not receive brachytherapy had 5.9 times more risk of mortality compared to those who had brachytherapy (aHR 5.9, *p*-value = 0.001). EQD2 ≥72 Gy was associated with 40% less mortality risk than those who had EQD2 of <72 Gy (aHR 0.4, *p*-value = 0.005). Patients with a maximum tumour diameter of more than 5 cm had 2 times higher mortality risk than those with a tumour with a maximum tumour diameter of less than 5 cm (aHR 2, *p*-value = 0.005). Patients who were treated with concurrent chemoradiotherapy had 60% less risk of mortality compared to those treated with radiotherapy alone (aHR 0.4, *p*-value = 0.048).

**Conclusion:**

UD was associated with a 53% reduction in baseline serum creatinine levels 30 days post-UD, reducing mortality risk by 40%.

## Background

Cervical cancer (CC) is the fourth most prevalent cancer for women worldwide, behind breast, colorectal and lung cancers, and occurs annually in 569,847 cases. It is also the fourth most common cause of cancer death (311,365 deaths in 2018) among women worldwide. CC develops in one in every 70 women worldwide between birth and age 79 [1].

Approximately 84% of CC cases and 88% of all CC deaths occur in low-income and low–middle-income countries, specifically due to the lack of routine screening and treatment [2]. In Sub-Saharan Africa (SSA), CC is the second leading cause of cancer-related deaths among women [2]. There are 18.8 million women over the age of 15 who are at risk for developing CC, and CC affects 10,241 women every year, with 6,525 of them dying from it in Tanzania, according to current estimates [3]. About 70% of women in SSA, including Tanzania, present with locally advanced stages of CC (LACC) at diagnosis [4–6].

Obstructive uropathy (OU) may develop when a blockage in the renal collecting system leads to distention of the renal calyces. Women with LACC often develop OU as a result of tumour or lymph node encroachment, inflammation or scarring at the pelvic rim [7]. In addition, CC is often treated with nephrotoxic drugs, which are sometimes dose-modified or omitted when OU is associated with renal insufficiency [7]. The incidence of OU in CC at presentation varies from 14% to 34.5% [8] and is often the ultimate cause of demise due to renal failure [9].

About 48% of patients with LACC with OU require urinary diversion (UD) before definitive treatment [7]. It is imperative to perform UD immediately, as long-term OU can result in intractable pain, infection and, ultimately, renal damage or failure, and OU is a poor prognostic sign in patients with LACC [7]. Three-year overall survival rates (OS) are 37% and 74%, respectively, for patients with LACC and without OU [7], with a median survival time of 3–12 months [10]. Due to this shorter life expectancy, most urologists are discouraged from performing reconstructive surgery. Therefore, palliative UD is given to many of these patients. Ureteral double J stent (DJS) and percutaneous nephrostomy (PCN) are two options for UD. UD can reduce patients’ suffering and protect renal function, which is essential for subsequent definitive treatment for LACC [11].

In Tanzania, PCN was started in 2018. Before October 2018, patients presenting with OU secondary to LACC were considered to be ineligible for concurrent chemoradiation therapy (CCRT) and were provided with symptomatic palliative care [12].

Although it may be intuitive to perform UD to normalise renal function and facilitate the delivery of radical CCRT, it is unclear whether such diversion procedures ensure the delivery of full-course radical CCRT and improve overall oncological outcomes [13]. Moreover, there are no clear guidelines for UD in patients with LACC. The recovery of renal functions and the benefits obtained from administering subsequent radiotherapy or chemotherapy are unpredictable. This study evaluated the benefits of UD by studying its effect on kidney function improvement 1-month post-UD and 3-year OS in women with LACC and OU in a resource-limited setting.

## Methods

### Study design

This retrospective bi-centered study used a secondary analysis of routine patient data from patients treated for LACC at Ocean Road Cancer (ORCI) Institute and Muhimbili National Hospital (MNH) in Dar es Salaam, Tanzania, from January 2020 to December 2021.

### Study site

ORCI and MNH are national referral, teaching and research hospitals providing care to women with LACC. MNH provides histological evaluation and conducts UD, including PCN and DJS. ORCI provides radiotherapy, chemotherapy and palliative care.

### Study population

Participants enrolled included those with (1) a confirmed histologic diagnosis of LACC (stage >3B), (2) OU as determined by renal ultrasound findings of hydronephrosis/hydroureter and (3) who could be reached by phone to answer the questionnaire. Patients with incomplete data and stage 4B disease were excluded.

### Variables

The primary dependent variable was a 3-year OS, and the secondary dependent variable was an improvement of renal function after UD. The independent variables included social demographic characteristics, clinical pathological characteristics, UD and treatment methods.

### Data collection

The medical charts were reviewed to obtain sociodemographic and clinical pathological data. Serum creatinine levels were obtained before the UD, 7 days after, 21 days after and 1 month after the UD. Information regarding the degree of OU and impairment of renal function was recorded. Data on co-morbid conditions and intercurrent medications that could negatively impact renal function were also gathered. Details were obtained about every UD procedure performed in this group, and their timing with radiotherapy was documented. Chemotherapy agents that were used were documented.

OS was defined as the duration from the completion date of treatment to loss to follow-up or death. Information about survival was obtained from the medical charts and through phone calls or other means of communicating with the patient or their next of kin. OU was diagnosed with an abdominal ultrasound.

### Statistical analysis

All data abstraction forms were checked for completeness before being entered into an MS Excel database and later analysed using R. The Shapiro–Wilk test was used to test if data was distributed normally. Normally distributed quantitative variables were summarised using mean and standard deviation, while non-normally distributed variables were summarised using median and interquartile ranges. Categorical variables were summarised using proportions. Student *t*-test was used to compare means, and the Mann–Whitney *U* test was used to compare medians. ANOVA statistical test with repeated measures was used to assess the trend of serum creatinine after UD. Survival was estimated using the Kaplan–Meier method. The prognostic value of the different variables for clinical outcome was calculated using a log-rank test in univariate analysis and the Cox proportional hazards regression model in multivariate analysis. The Schoenfeld residual test was used to test whether proportional hazard assumptions were met. A competing risk analysis was done using the Fine and Gray Model to estimate the cumulative incidence function (CIF) for patients who have died and those who were lost to follow-up. Gray’s test was done to compare CIFs.

The formula used to estimate Cohen’s d (d) from hazard ratio (HR) and proportion of events in the control group (P_0_) was d = ln (HR)/√ (1/P_0 +_ (1/1-P_0_)) [14].

The significance level was set at 0.05.

### Ethical consideration

Ethical clearance for conducting this study was sought from the Muhimbili University of Health and Allied Sciences (MUHAS) Ethical Review Board (approval number: MUHAS-REC-04-2024-2170). The ORCI Ethical Review Board approved the study. Because of the study’s retrospective nature, the MUHAS ethics review board waived informed consent.

## Results

Of 2,226 patients with CC, we extracted data from the medical charts of 1,215 patients with LACC treated between January 2020 and December 2021 at MNH and ORCI. Of these 1,215 patients, 167 met the OU and hydronephrosis eligibility criteria. Of these 167 patients, 119 met the inclusion criteria for this study. Approximately one third of these 119 patients had UD. Bilateral PCN was the most frequently performed type of UD (92%). Among the patients who underwent UD (*n* = 37), 76% had it before and 24% had it after radiotherapy.

The mean age was 51.1 ± 9.9 years. Only about 15% of the participants had health insurance. Most patients with health insurance underwent UD (64%), while only 38% of patients without health insurance underwent UD (*p* = 0.003) (Table 1).

HIV (31.1%) and hypertension (13.4%) were the most common comorbidities in patients. Patients who underwent UD had a higher median ECOG score than those who did not (*p* = 0.00) (Table 2).

The majority of patients had hydronephrosis (85.7%). About 35% of patients with hydronephrosis underwent UD, and only 5.9% without hydronephrosis underwent UD (*p* = 0.02). The majority of the patients had unilateral hydronephrosis (56.3%). Only 22% of patients with unilateral hydronephrosis underwent UD, while 47.3% of patients with bilateral hydronephrosis underwent UD (*p* = 0.02). Patients with unilateral hydronephrosis outnumber those with bilateral hydronephrosis by 56.3% to 47.3%. Patients with moderate and severe hydronephrosis were the most common, accounting for 77.3.% % of all cases. Most of the patients who had severe hydronephrosis (45%) underwent UD, while only a few of the respondents with mild OU underwent UD (18.5%) (*p* = 0.045). Patients who underwent UD had a higher mean serum creatinine (707 µmol/L) than those who did not (402 µmol/L) (*p* = 0.001) (Table 3).

About 23.5% of patients underwent UD before RT, while for 68% of patients, it was not known whether UD was done before or after RT. Among those who were given chemotherapy, the most common agent was Cisplatin (94%). The median baseline serum creatinine among patients treated with CCRT was 182 µmol/L; for those treated with RT alone, it was 527 µmol/L (*p* = 0.000). 43 (36.1%) respondents were treated with CCRT alone without UD. The median number of weekly chemotherapy cycles was 3 (Table 4).

**Figure 1.**
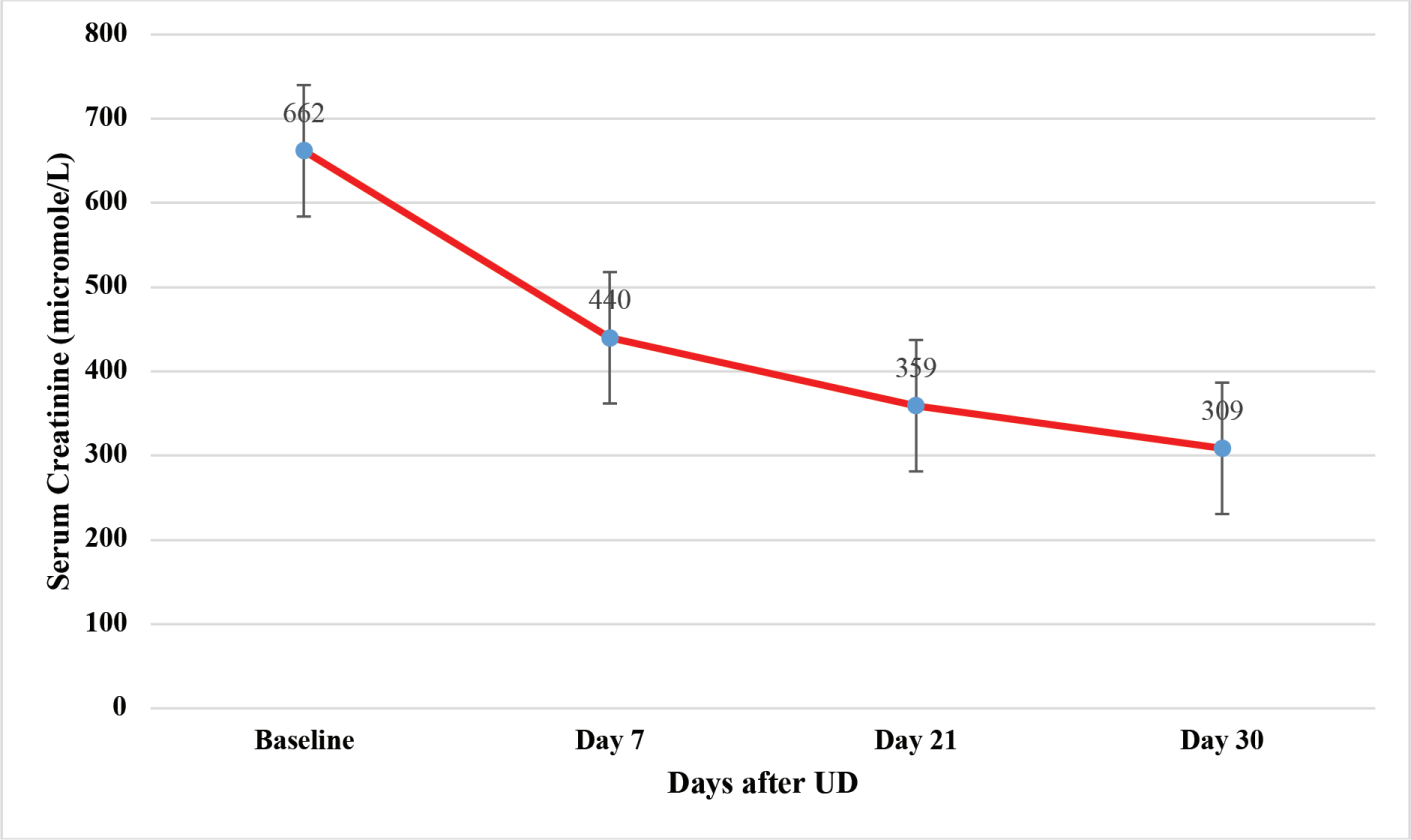
The trend of serum creatinine after UD.

The baseline serum creatinine of women who underwent UD decreased by 53% (*p* = 0.002) (Figure 1).

**Figure 2.**
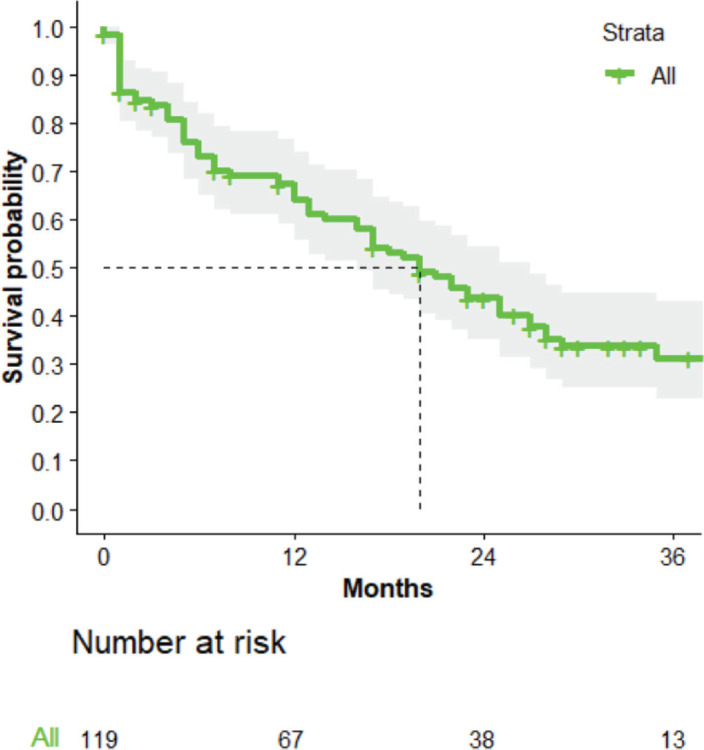
The OS curve for the entire cohort.

The median survival time was 20 months (Figure 2).

**Figure 3.**
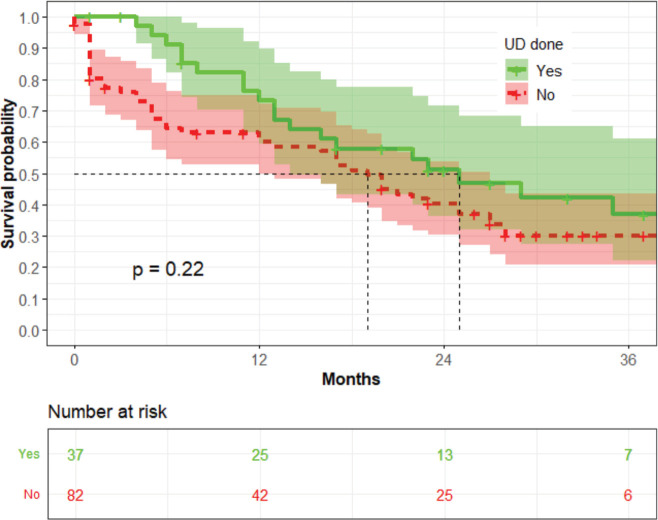
The association between UD and OS (*n* = 119).

The median survival time among women who underwent UD was 25 months, while that of those who did not was 16 months (*p* = 0.22) (Figure 3). 

The CIFs for women who underwent UD and those who did not undergo UD were not statistically different for death (*p* = 0.51) or due to loss to follow-up (LTF) (*p* = 0.14) (Table 5).

**Figure 4.**
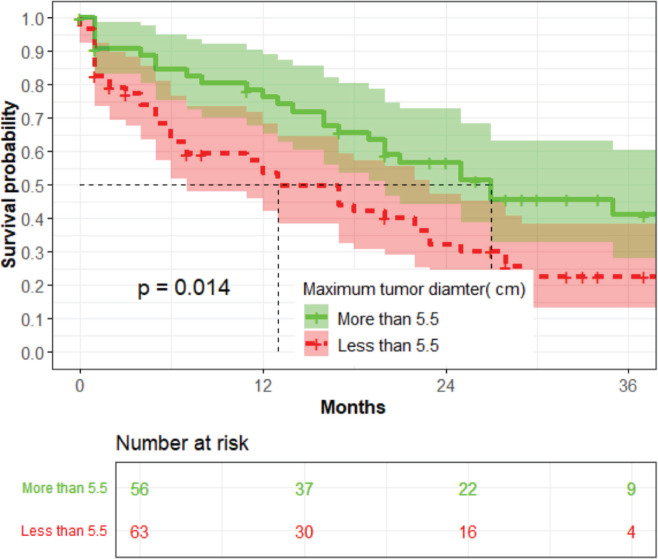
The association between the maximum diameter of the tumour and OS (*n* = 119).

The median survival time for those with a tumour diameter greater than 5 cm was 13 months, while that for those with a tumour smaller than 5 cm was 26 months (*p* = 0.014) (Figure 4).

**Figure 5.**
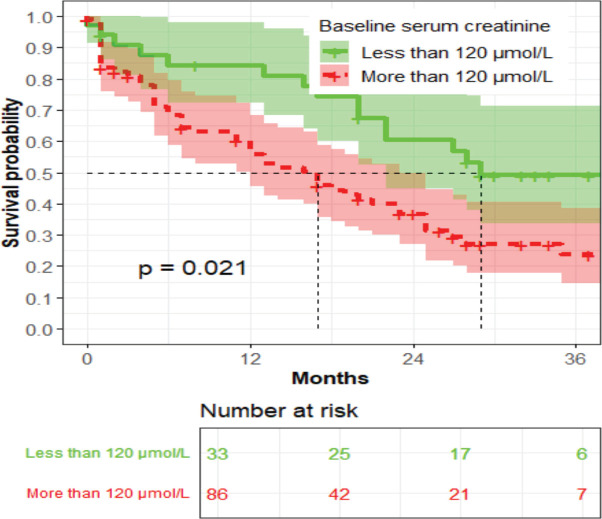
The association between baseline serum creatinine and OS (*n* = 119).

The median survival time among women whose baseline serum creatinine was above 120 µmol/L was 19 months, while that of women with baseline serum creatinine below 120 µmol/L was 27 months (*p* = 0.021) (Figure 5).

**Figure 6.**
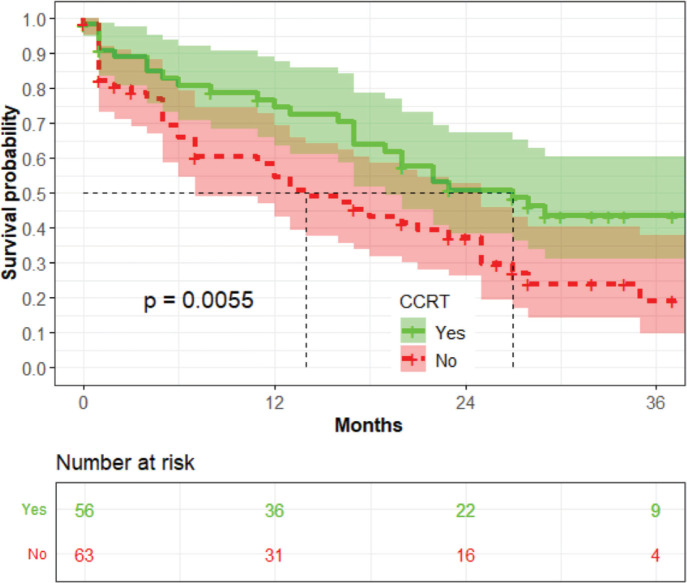
The association between the use of CCRT and OS (*n* = 119).

Women treated with CCRT had a higher median survival time than those treated with radiotherapy alone (*p* = 0.0055) (Figure 6).

**Figure 7.**
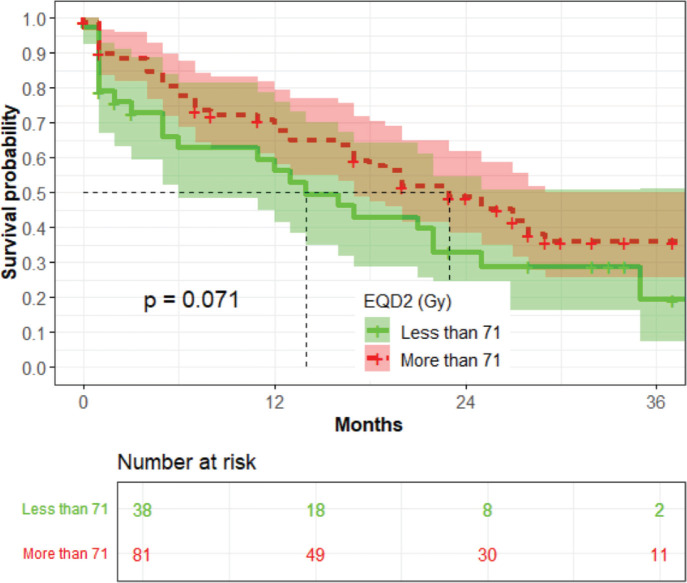
The association between the EQD2 and OS among (*n* = 119).

In the univariate analysis, the equivalent dose in 2-Gy (EQD2) was not associated with OS (*p* = 0.071) (Figure 7).

**Figure 8.**
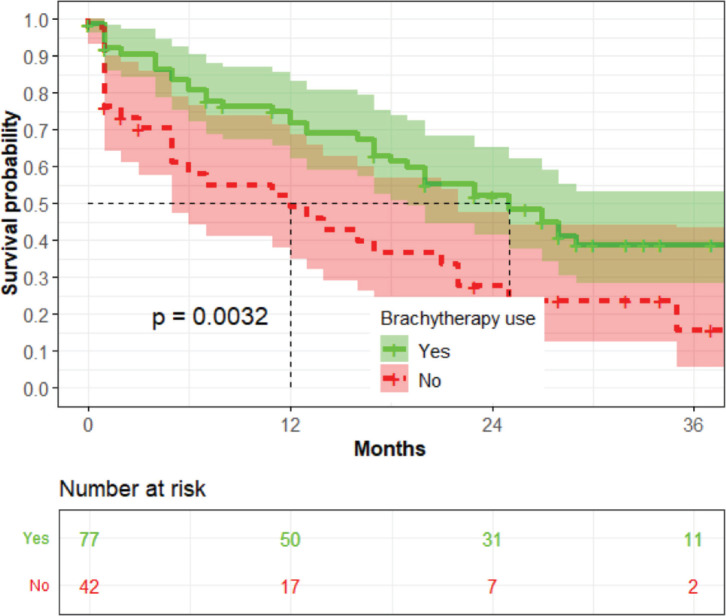
The association between the brachytherapy use and OS (*n* = 119).

The women who received brachytherapy had a median survival time of 24 months, while those who did not had a median survival time of 12 months (*p* = 0.0032) (Figure 8).

The covariates associated with a low mortality risk were UD, tumour size of less than 5 cm, baseline serum creatinine of less than 260 µmol/L, unilateral hydronephrosis and use of brachytherapy (Table 6).

**Figure 9.**
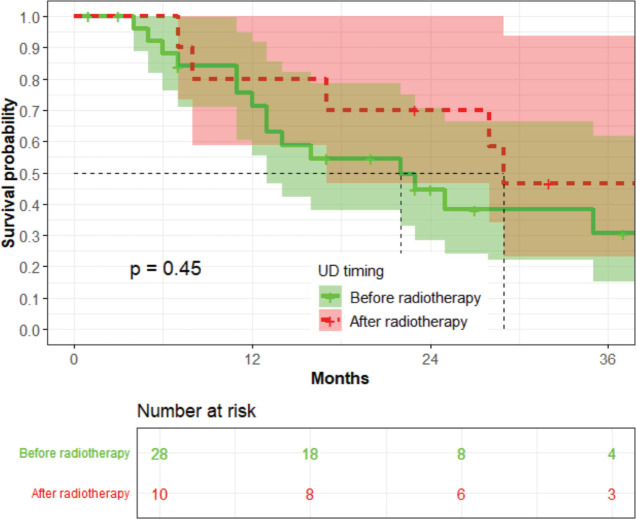
The association between the timing of UD and OS among patients who underwent UD (*n* = 38).

The timing of UD did not influence OS (*p* = 0.45) (Figure 9).

Women with tumours larger than 5 cm were 2 times more likely to die compared to those with tumours smaller than 5 cm (*p* = 0.03). Those who did not receive brachytherapy were 5.9 times more likely to die than those who did (*p* = 0.001). The risk of dying was 1.9 times greater for patients with a baseline serum creatinine of more than 260 µmol/L compared to those with a baseline serum creatinine of less than 260 µmol/L. The mortality risk of women treated with EQD2 doses greater than 72 Gy decreased by 60% compared to those treated with lower doses (*p* = 0.048). Those who underwent UD had a 40% lower mortality risk than those who did not (*p* = 0.03) (Table 7).

The Cohen’s D was estimated to be 0.3, indicating a small effect size.

There was no violation of the proportional hazard assumption (*p* = 0.16) (Table 8).

**Figure 10.**
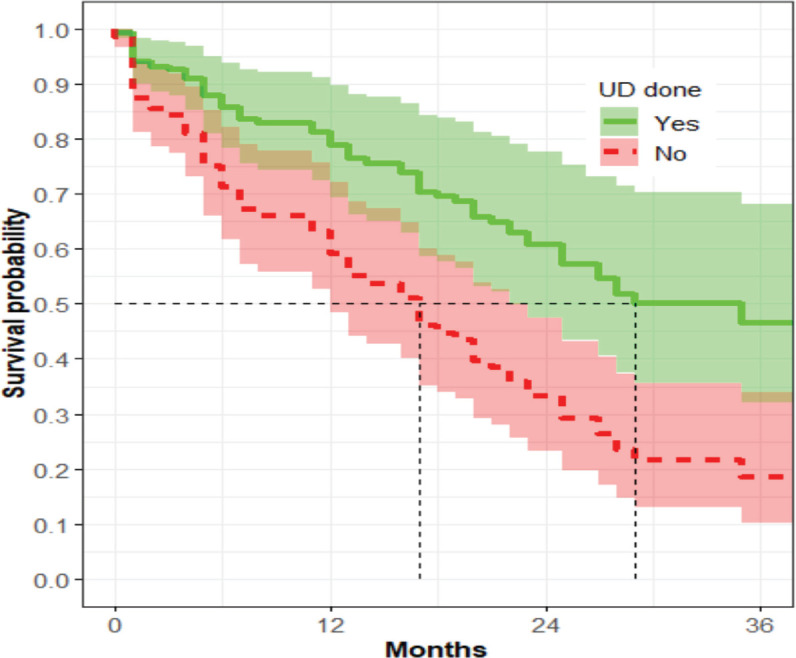
The adjusted survival curves.

After adjusting for tumour size, EQD2, brachytherapy use and baseline serum creatinine, women who underwent UD had a median survival time of 16.5 months, while those who did not undergo UD had a median survival time of 28.5 months (*p* = 0.03) (Figure 10).

## Discussion

We have examined many aspects of the clinical outcome of women with LACC with OU before and after UD. To the best of our knowledge, this study is the first to explore the impact of UD on OS among women with LACC treated with definitive radiotherapy in SSA.

This study’s most common UD type was PCN (92%). OU can result from an intrinsic source, such as stones or extrinsic compression, such as LACC. Stent placement has been well-documented to relieve almost all obstructions due to intrinsic pathology, but only 50% of obstructions are caused by extrinsic compression. Predictors of stent failure include the cancer diagnosis, baseline serum creatinine greater than 114 umol/L and post-stent therapy necessitating radiation or chemotherapy [15]. Furthermore, a CT or MRI finding of ureteral obstruction >3 cm in length is another significant risk factor for ureteral stent placement failure and conversion to PCN [11]. However, compared to PCN, ureteral stent placement has a significantly lower cost and shorter surgical and hospitalisation time [11].

The study showed that 64% of patients with health insurance underwent UD, while only 38% of patients who did not have health insurance underwent UD (*p* = 0.003). Access to UD was lower for patients who did not have health insurance. A cross-sectional study in Tanzania found that the unit cost of providing the newly introduced PCN to LACC patients with hydronephrosis was USD 380.4. This procedure is costly in a nation where per capita health expenditure is USD 40.5, and out-of-pocket health expenditure accounts for 32% of total health expenditure. According to estimates, PCN’s unit cost is three times as much as the minimum monthly government wage and more than four times as much as in the private sector [16].

The extent and duration of OU are the main factors determining how well renal function recovers after UD [17]. According to Horan *et al* [8], patients with bilateral hydronephrosis and a low (less than 50 mL/min (0.84 mL/s) creatinine clearance, which may correspond to serum creatinine of approximately 120 µmol/L approximately should be considered for UD before starting radiotherapy and pelvic radiation does not induce any deterioration of renal function or degree of hydronephrosis. The baseline median serum creatinine in the index study was 216 µmol/L, underscoring the severity of OU among these patients. Women who underwent UD had a higher mean serum creatinine (707 µmol/L) before the procedure than those who did not, indicating a delay in performing UD in this study. OU lasting longer than 4 to 6 weeks is usually regarded as irreversible, although partial recovery and stopping dialysis have been reported even after 7 months of complete obstruction [18]. Women who have LACC and bilateral hydronephrosis and a baseline serum creatinine of more than 120 µmol/L may require early UD before definitive radiotherapy. The timing of UD did not influence OS in the index study, perhaps because it was conducted late when OU was already advanced.

For all patients who had PCN in this study, it was done bilaterally. Hyppolite *et al* [19] in their study of OU in gynecological malignancies, found bilateral nephrostomy to be superior in improving renal function to unilateral nephrostomy and even to intraureteric stenting. They advocated against placing intraureteric stents in LACC patients, as it was linked to an 86% chance of urosepsis and a death rate of 43% [19]. However, a study by Dhani *et al* [20] showed that the survival rate did not differ significantly between ureteral stents (median survival 11 months) and PCN (median survival 15 months) (*p* = 0.74). Similar findings were observed in a systematic review and meta-analysis done by Gauhar *et al* [21] comparing PCN and ureteral stenting.

A month after UD, the renal function of patients who had UD improved significantly by 53%, with the mean serum creatinine dropping from 662 to 309 µmol/L (*p* = 0.002). This finding corresponds with one study done by Eder *et al* [22] and another one done at ORCI and MNH by Rukundo *et al* [12], who reported 3- and 10-days post UD, the mean creatinine levels improved from 444.65 to 163.54 µmol/L, respectively [12]. Patient survival is enhanced by complete renal recovery on the 30th day after UD [23]. However, the improvement in renal function after UD is usually short-lived, requiring the patient to return to dialysis after a short period [20]. The non-recovery of renal function after the UD also depends on age and renal cortical thickness. Age beyond 50 years and decreased renal cortical thickness (less than 13 mm) indicate poor recovery of renal function [17]. A study by Candelaria *et al* [24] was conducted on nine women with LACC presenting with OU and treated with pelvic radiotherapy concurrently with weekly gemcitabine at 300 mg/m^2^ showed that eight (89%) of the nine patients achieved complete response, and one patient had stable disease. All patients were alive at a median follow-up of 11 months [24]. A meta-analysis by Vale [25] showed a significant benefit associated with non-platinum radiosensitisers.

Despite the improvement in renal functioning among patients who had UD, only 32% of them received CCRT, while 52% of patients who did not have UD received CCRT. The low rate of CCRT used among patients with UD could be due to higher baseline serum creatinine (502 µmol/L) compared to 200 µmol/L among patients who did not undergo UD, and possibly also due to lack of awareness of other less nephrotoxic radiosensitisers apart from cisplatin. The most common radiosensitiser used in the index study was cisplatin (94%). Cisplatin’s high nephrotoxic effects and high risk of worsening preexisting renal failure make it unsuitable for use as a radiosensitiser among women with LACC and OU. Carboplatin and Gemcitabine are among the alternatives less nephrotoxic radiosensitisers [14].

In the univariate analysis, UD was not associated with OS; however, in the multivariate analysis, UD was associated with a 40% decreased risk of mortality (aHR 0.6; *p* = 0.03). Non-significance in univariate analysis but significance in multivariate analysis in this study could be due to four possible scenarios in this study: (1) the effect of unbalanced sample size; (2) the influence of missing data; (3) an extremely large within-group variation, relative to between-group variation and (4) the presence of interaction [25]. The reason for the change from non-significant in univariate analysis to significant in multivariate analysis can be linked to controlling confounding factors, interactions, increased statistical power and improved model specification. To date, it remains unclear whether UD is beneficial in patients with malignant OU [26]. A retrospective study by Nugroho *et al* [27] also showed that UD improved OS among patients with LACC in Indonesia. A prospective study by Lapitan and Buckley [28] in the Philippines showed UD improved survival 3 months after the procedure among women with LACC who underwent UD compared to those who did not undergo the procedure; however, at 12 months, there was no difference between the two groups. More studies are needed to evaluate the role of UD in this group.

Women treated with CCRT had a longer median survival time than those treated with radiotherapy alone (*p* = 0.0055). Our study shows that CCRT is still beneficial among women with LACC. Systematic reviews have revealed that CCRT is better suited to treating LACC than RT alone [25]. As stages increase, the relative effect of CCRT on survival is thought to diminish, with estimated absolute 5-year survival benefits of 10% at stages Ia–IIa, 7% at stages IIb and 3% at stages III-Iva [25]. There could be room for improvement in CCRT as a standard treatment for stages III–IV patients.

Using brachytherapy and EQD2 of more than 72 Gy to point A was associated with prolonged survival, underscoring the need to treat patients with LACC and OU radically. External beam radiation therapy (EBRT) and brachytherapy are the standard definitive treatments for LACC. Brachytherapy is necessary to deliver a highly effective dose to the primary tumour: more than 80–85 Gy biologically EQD2 can routinely be delivered to the tumour periphery while the central cervix receives even higher doses (>120 Gy EQD2) [29]. In a study by Tanderup *et al* [29], women with LACC treated with combined EBRT and brachytherapy had significantly better OS than those treated with EBRT alone (65% and 50%, respectively).

In a pre-post retrospective study design using the SF-12 tool by Texeira *et al* [30], the quality of life of women with OU improved significantly at 1 week (*p* = 0.000) and 1 month (*p* = 0.000), which was not sustained at the third month (*p* = 0.219). In another pre-post retrospective study design by Kiende *et al* [9], PCN had an overall improvement in the quality of life (FACT-Cx overall *p* = 0.041) with significant changes reported in aspects of physical well-being (*p* = 0.018) and additional concerns such as sexual function, self-esteem and appearance, urinary function, appetite and gastrointestinal function (*p* ≤ 0.001). A prospective study by Lapitan and Buckley [28] among women with LACC and OU concluded that quality of life scores did not significantly differ across groups with UD and that without UD over the entire study period [28]. More prospective studies with controls are needed to evaluate the role of UD in improving the quality of life of women with LACC.

Some of this study’s limitations include its limited sample size with many missing data, retrospective methodology, lack of health-related quality-of-life data and morbidity data associated with UD and high rate of loss to follow-up (16%). Future research should involve a prospective study to include more participants to validate our findings and evaluate the quality of life associated with UD among patients with LACC.

In summary, this study adds to the extant published literature on OU in patients with LACC and OU. It demonstrates that UD significantly improved post-UD serum creatinine levels by 53% and decreased mortality risk by 40% among these patients.

## List of abbreviations

CC, Cervical cancer; CCRT, Concurrent chemoradiation therapy; LACC, Locally advanced cervical cancer; LINAC, Linear accelerator; OS, Overall survival; OU, Obstructive uropathy; PCN, Percutaneous nephrostomy; SSA, Sub-Saharan Africa; UD, Urinary diversion.

## Conflicts of interest

The authors declare no competing interests.

## Funding

This research received no external funding.

## Consent for publication

Not applicable.

## Author contributions

Conception and design: GLS, LAR and ELL

Financial support: GLS

Provision of study materials or patients: GLS and ELL

Data collection and assembly: GLS and ELL

Data analysis and interpretation: GLS and EL

Manuscript writing: All authors

Final approval of manuscript: All authors

Accountable for all aspects of the work: All authors

LM and EL are the first authors with equal contributions.

## Availability of data and materials

The data presented in this study are available on reasonable request from the corresponding author.

## Figures and Tables

**Table 1.** Social demographic characteristics of respondents.

Variable		Entire cohort	UD		*p*-value
			Yes	No	
Age (Years)	Mean ( ± SD)	51 ± 9.9	49.9 ± 10.4	51.7 ± 10.4	0.37
Alcohol	Yes	23 (19.3)	4 (17.4)	19 (82.6)	0.11
	No	96 (80.7)	33 (34.4)	63(65.6)	
Smoking	Yes	8(6.7)	2 (25.0)	6 (75.0)	0.7
	No	111 (93.3)	35 (31.5)	76 (68.5)	
Insurance	Yes	18 (15.1)	11 (61.1)	7 (38.9)	**0.003**
	No	101 (84.9)	26 (25.7)	75 (74.3)	

IQR- Interquartile range

**Table 2.** Clinical pathological characteristics of patients.

Variable		Entire cohort	UD		*p*-value
			Yes	No	
HIV	Positive	37 (31.1)	9 (24.3)	28 (75.7)	0.23
	Negative	65 (56.6)	20 (30.8)	45 (69.2)	
	Unknown	17 (14.3)	8 (47.1)	9 9 (52.9)	
ECOG score	Median (IQR)	1 (0–3)	1 (1–2)	1(1–3)	**0.00**
Hemoglobin levels (g/dL)	Mean ( ± SD)	8.7 ± 2.4	8.7 ± 2.4	8.6 ± 2.4	0.91
Hypertension	Yes	16 (13.4)	8 (50.0)	8 (50.0)	0.36
	No	88 (74.0)	24 (27.3)	64 (72.7)	
	Unknown	15 (12.6)	5 (33.3)	10 (66.7)	
Maximum tumor diameter (cm)	Mean ( ± SD)	**5.7 ± 1.7**	5.5 ± 1.7	5.8 ± 1.7	0.25

**Table 3.** Renal functioning characteristics of patients.

Variable		Entire cohort	UD		*p*-value
			Yes	No	
Hydronephrosis	Yes	102(85.7)	36 (35.3)	66 (**No** 66.7)	**0.02**
	No	17 (14.3)	1 (5.9)	16 (94.1)	
Hydronephrosis site	Unilateral	67 (56.3)	15 (22.4)	52(77.6)	**0.02**
	Bilateral	52 (47.3)	22 (42.3)	20 (57.7)	
Hydronephrosis grade	Mild	27 (22.7)	5 (18.5)	22 (81.5)	0.045
	Moderate	52 (43.7)	14 (26.9)	38 (73.1)	
	Severe	40 (33.6)	18 (45.0)	22 (55.0)	
Baseline serum creatinine	Median (IQR)	216 (105–24,54)	506 (105–2,454)	200 (120–2,000)	**0.002**

IQR- Interquartile range

**Table 4.** Treatment patterns of patients with LACC with OU and/or hydronephrosis.

Variable		Entire cohort	UD		*p*-value
			Yes	No	
CCRT	Yes	56 (47.1)	12 (21.4)	44 (78.6)	**0.03**
	No	63 (52.6)	25 (39.7)	38 (60.3)	
Treatment patterns	UD & CCRT	12 (10.1)	12 (100.0)	0 (0)	**0.00**
	CCRT alone	43 (36.1)	0 (0.0)	43(100.0)	
	UD & RT	25 (54.6)	25 (0.0)	0 (0.00)	
	RT alone	39 (32.8)	0(0.00)	39 (100.0)	
EBRT technique	2D	83 (69.7)	5 (18.5)	22 (81.5)	**0.045**
	3DCRT	33 (27.7)	14 (26.9)	38 (73.1)	
Chemotherapy cycles	Median (IQR)	3 (1–5)	3 (1–5)	3 (1–5)	0.82
EQD2 (Gy)	Median (IQR)	86 (33–86)	86 (33–86)	86 (33–86)	0.26
Brachytherapy	Yes	78 (65.5)	23 (29.5)	55 (70.5)	0.6
	No	41 (34.5)	14 (34.1)	72 (65.9)	

**Table 5.** Competing risk analysis: the results of Gray’s test for equality of CIFs across patients who underwent UD and those who did not undergo UD.

Competing events	Statistic	*p*-value	df
Death	1.06	0.51	1
LTF	2.13	0.14	1

LTF- Loss to follow up: df-degree of freedom

**Table 6.** Summary of univariate analysis.

Variable		Univariate analysis		
		*p*-value	cHR	95% CI
UD	Yes	0.23	0.7	0.4–1.2
	No	1		
ECOG	≥1	0.07	1.7	0.9–2.1
	0	1		
Tumor size (cm)	>5	0.02	2.17	1.1–2.9
	<5	1		
Baseline serum creatinine (micromoles/L )	>260	0.02	1.8	1.1–2.9
	<260	1		
Hydronephrosis	No	0.06	0.4	0.2–1
	Yes	1		
Hydronephrosis type	Bilateral	0.008	1.9	1.1–3
	Unilateral	1		
Hydronephrosis grade	Moderate & Severe	0.002	3.0	1.5–6.5
	Mild	1		
EQD2 (Gy)	>72	0.08	0.6	0.4–1
	<72	1		
Brachytherapy use	No	0.005	2	
	Yes	1		
CCRT use	Yes	0.2	2.6	0.6–10
	No	1		
HIV status	Positive	0.5	1.2	0.7–2
	Negative			
Health insurance	Yes	0.6	1.2	0.6–2.3
	No			

cHR-Crude Hazard Ratio

**Table 7.** Summary of multivariate analysis.

Variable		Multivariate analysis		
		*p*-value	aHR	95% CI
UD	Yes	0.03	0.6	0.3–0.9
	No	1		
Tumor size (cm)	>5	0.005	2.0	1.2–3.3
	<5	1		
CCRT	No	0.003	2.3	1.3–4.0
	Yes	1		
EQD2 (Gy)	>72	0.048	0.4	0.1–0.9
	<72	1		
Brachytherapy use	No	0.001	5.9	2.1–16.5
	Yes	1		

aHR-Adjusted Hazard Ratio

**Table 8.** A summary of the Schoenfeld residual test.

Covariate	Chi-square	df	*p*-value
UD	6.78	1	0.009
Tumor size	0.46	1	0.497
CCRT	1.52	1	0.219
EQD2	0.003	1	0.958
Brachytherapy use	0.06	1	0.737
Global	8.83	5	0.150

df-degree of freedom; EQD2- Equivalent dose in 2 Gy Fractions
